# Gpr97 Is Dispensable for Inflammation in OVA-Induced Asthmatic Mice

**DOI:** 10.1371/journal.pone.0131461

**Published:** 2015-07-01

**Authors:** Jue-ping Shi, Xiao-ning Li, Xiao-yu Zhang, Bing Du, Wen-zheng Jiang, Ming-yao Liu, Jin-jin Wang, Zhu-gang Wang, Hua Ren, Min Qian

**Affiliations:** 1 Shanghai Key Laboratory of Regulatory Biology, School of Life Sciences, East China Normal University, Shanghai, China; 2 Institute of Biomedical Sciences, School of Life Sciences, East China Normal University, Shanghai, China; 3 Shanghai Research Center for Model Organisms, Shanghai, China; Université Libre de Bruxelles, BELGIUM

## Abstract

**Background:**

Asthma is a complex inflammatory disorder involving the activation and invasion of various immune cells. GPR97 is highly expressed in some immunocytes, including mast cells and eosinophils, which play critical roles in asthma development. However, the role of Gpr97 in regulating airway inflammation in asthma has rarely been reported. In this study, we investigated the potential role of Gpr97 in the development of allergic asthma in mice.

**Methods:**

Relevant airway asthmatic mouse models were constructed with both wild-type and *Gpr97^-/-^* mice sensitized to 250 μg ovalbumin (OVA). The levels of interleukin IL-4, IL-6 and IFN-γ, which are involved in OVA-induced asthma, in the bronchoalveolar lavage fluid (BALF) and the IgE level in the serum were examined by enzyme-linked immunosorbent assay (ELISA). The invasion of mast cells and eosinophils into lung tissues was assessed by immunohistochemical and eosinophil peroxidase activity assays, respectively. Goblet cell hyperplasia and mucus production were morphologically evaluated with periodic acid-Schiff (PAS) staining.

**Results:**

In our study, no obvious alteration in the inflammatory response or airway remodeling was found in the *Gpr97*-deficient mice with OVA-induced asthma. Neither the secretion of cytokines, including IL-4, IL-6 and IFN-γ, nor inflammatory cell recruitment was altered in the *Gpr97*-deficient mice. Moreover, *Gpr97* deficiency did not affect airway remodeling or mucus production in the asthma mouse model.

**Conclusion:**

Our findings imply that Gpr97 might not be required for the development of airway inflammation in OVA-induced allergic asthma in mice.

## Introduction

Asthma is one of the most common chronic lung diseases, and it is characterized by reversible airway obstruction, airway hyperresponsiveness, and airway inflammation [1–3]. Most cases of asthma arise from sensitization of the airways to aeroallergens, such as house dust mites, animal dander, fungi and cockroaches [4]. Allergic airway inflammation, including the innate and adaptive immune responses, is a striking feature of asthma and is assumed to be the initiating event of airway remodeling [5,6]. Different immune cells, such as eosinophils, macrophages, T helper type 2 (Th2) cells, and mast cells, are involved in the inflammatory response of asthma [5]. An early inflammatory disorder of the pulmonary airway is involved in dendritic cell-dependent Th2 cell maturation and Th2 cell-dependent IgE production by B cells [7]. Meanwhile, some cytokines are additionally secreted to promote the activation and invasion of mast cells and eosinophils. These two types of immune cells can increase inflammatory reactions by releasing cytokines and regulate airway remodeling by promoting mucus hypersecretion and airflow limitation during the asthma process.

It has been widely reported that G protein-coupled receptors (GPCRs) can mediate a variety of cellular responses induced by extracellular signals, including inflammatory reactions [8]. As a subpopulation of GPCRs, adhesion GPCRs play critical roles in the central nervous system, the immune system and tumor formation [9,10]. GPR97, a type of adhesion GPCR, has been demonstrated to be over-expressed in lymphatic endothelial cells, mast cells and eosinophils [10,11]. Mast cells and eosinophils are two important cells involved in inflammatory responses in allergic asthma. However, the functions of GPR97 in mast cells and eosinophils have not been elucidated to date. Recently, Gpr97 has been found have an important role in regulating B cell development in mice [12]. B cells are important immune cells that are responsible for IgE production in asthma [13]. Theses findings indicate that Gpr97 might have a function in the inflammatory response of allergic asthma.

In the present study, we developed ovalbumin (OVA)-induced airway inflammation mouse models in wild-type (WT) and *Gpr97*-deficient mice and examined the severity of the inflammatory response and airway alterations that take place during asthma. Features of asthma, such as the release of cytokines, immune cell invasion and airway remodeling, were measured in *Gpr97*-deficient mice following OVA challenge. The aim of our research was to clarify the potential function of Gpr97 in allergic asthma in mice. However, our results suggested that *Gpr97* deficiency did not alter the airway inflammatory response and remodeling in the OVA-induced asthmatic mouse model, indicating that GPR97 might not be essential to the process of allergic asthma.

## Materials and Methods

### Animals

Wild-type (WT) C57BL/6 mice were obtained from the laboratory animal center of East China Normal University (Shanghai, China). *Gpr97* ^-/-^ C57BL/6 mice were donated by the Dr. Zhu-gang Wang Laboratory (Shanghai Research Center for Model Organisms, Shanghai, China). All mice were bred and housed under pathogen-free conditions and maintained according to institutional guidelines. The mice were 6–8 weeks old at the time of the experiments. All mice were sacrificed using the method of dislocation of vertebrate before experiment. All experimental protocols were approved by the Animal Investigation Committee of East China Normal University. Age-matched littermates (6–8 weeks old) with different genotypes were used for OVA-induced asthmatic mice models. Before the experiments, *Gpr97* deficiency in the mice was confirmed by PCR [12].

### OVA sensitization and challenge in mice

For the construction of an suitable asthma mouse model [14], a sensitization concentration of 50 μg, 100 μg or 250 μg ovalbumin (OVA, grade V, Sigma-Aldrich, St. Louis, USA) plus 1% Al(OH)_3_ in a volume of 200 μl PBS was intraperitoneally injected into different groups of WT mice on days 0, 7 and 14. On days 21 to 27, sensitive mice were challenged by atomization with 1% OVA in PBS or PBS only for 30 min each time. At 24 hours after the last challenge, all mice were euthanized, and serum, bronchoalveolar lavage fluid (BALF) and lung tissues were collected. The *Gpr9*7-deficient mice were sensitized by intraperitoneal injection with the optimal sensitization concentration of OVA according to the former assessment, and control WT asthma mice were induced under the same conditions at the same time.

### Bronchoalveolar lavage fluid leukocyte count

Lungs were flushed twice with cold 0.5% fetal bovine serum in 1 mL PBS. BALF was obtained after lavage and centrifuged at 2000 g for 5 min at 4°C. The depositions were resuspended in 50 μL PBS, and the total number of cells was counted with a hemocytometer.

### Cytokine and IgE analysis

Blood samples were centrifuged at 3000 g for 10 min at 4°C to obtain serum samples, which were used to detect the levels of IgE with a commercial enzyme-linked immunosorbent assay (ELISA) kit (Biolegend, California, USA). The levels of interleukin IL-4, IL-6 and IFN-γ in the BALF were detected using the corresponding ELISA kit following manufacturer’s instructions.

### Eosinophil peroxidase activity (EPO)

Right upper lung tissue from each sample was homogenized in PBS (0.01 g/100 μL) and frozen and thawed three times in liquid nitrogen. The samples were centrifuged at 12000 g for 20 min at 4°C to collect the supernatants. A total of 75 μL of supernatant was mixed with 150 μL Tris-HCl (pH 8.0) containing 1.5 mM o-phenylenediamine (Sigma) and 6.6 mM H_2_O_2_ (Sinopharm Chemical Reagent Co., Ltd., Beijing, China), followed by incubation for 30 min at room temperature, according to the instructions of the kits. A total of 75 μL of 30% H_2_SO_4_ was used to stop the reaction, and the absorbance was read at a 492 nm wavelength [15,16].

### Histology and immunohistochemistry

To analyze goblet cell hyperplasia, the left upper lung from each mouse was fixed in 4% paraformaldehyde overnight (24 h), embedded in paraffin and cut into 5-μm sections. The slices were dewaxed in dimethylbenzene and rehydrated in decreasing concentrations of ethanol. Before being stained with periodic acid-Schiff (PAS) reagent, the slices were incubated in periodic acid alcohol. Then, they were washed with sulfurous acid, followed by nucleolus staining using hematin and mounting in glycerol. PAS-positive cells and mucus secretion were observed under a light microscope.

The left lower lung tissues were fixed in 4% paraformaldehyde overnight and then dehydrated in 20% and 30% sucrose, followed by embedding in OTC medium (Sakura, Finetek, USA). Ten-micrometer-thick tissue slices were prepared using the frozen section method, and they were then washed with PBS and incubated with a CD117 antibody (1:50, eBioscience, San Diego, USA) at 4°C overnight. Next, CD117-positive mast cells were stained using an immunohistochemistry kit (Zhongshanjinqiao, Beijing, China).

Before observing subepithelial fibrosis in the lungs, the lung tissue slices were incubated in periodic acid alcohol. Then, slices were washed with deionized water, followed by nucleolus staining using hematoxylin and followed by Masson’s trichrome-stained reagent (Zhongshanjinqiao, Beijing, China). The slices were observed under a light microscope after mounting in glycerol [17].

### OVA sensitization and challenge on cell

After the primary separating of bone marrow cells from wild-type mouse, IL-3 and SCF (Biolegend, California, USA)-induced differentiation was used to get purified mast cells. Then the flow cytometry analysis was used to separate CD117 and FcεRIα positive mast cells (APC-CD117 and FITC-FcεRIα, Biolegend, California, USA). Serum containing IgE from OVA-induced mice was used to activate purified mast cells overnight. Mast cells were challenged with 200μg OVA for 1h, 3h and 6h separately. Then, Real-time PCR was used to detect the mRNA expression level of Gpr97 on mast cells.

### Statistical analysis

Significant differences were assessed with the *t*-test for comparisons between two groups and ANOVA or ANOVA+Tukey test for comparisons among more than two groups of mice. The data are presented as the mean ± SEM. All statistical values were measured using GraphPad Prism version 5.0 (GraphPad Prism Software, USA). Value of *P*< 0.05 was considered to be significant.

## Results

### OVA-induced allergic asthma mouse model construction

**Fig 1:**
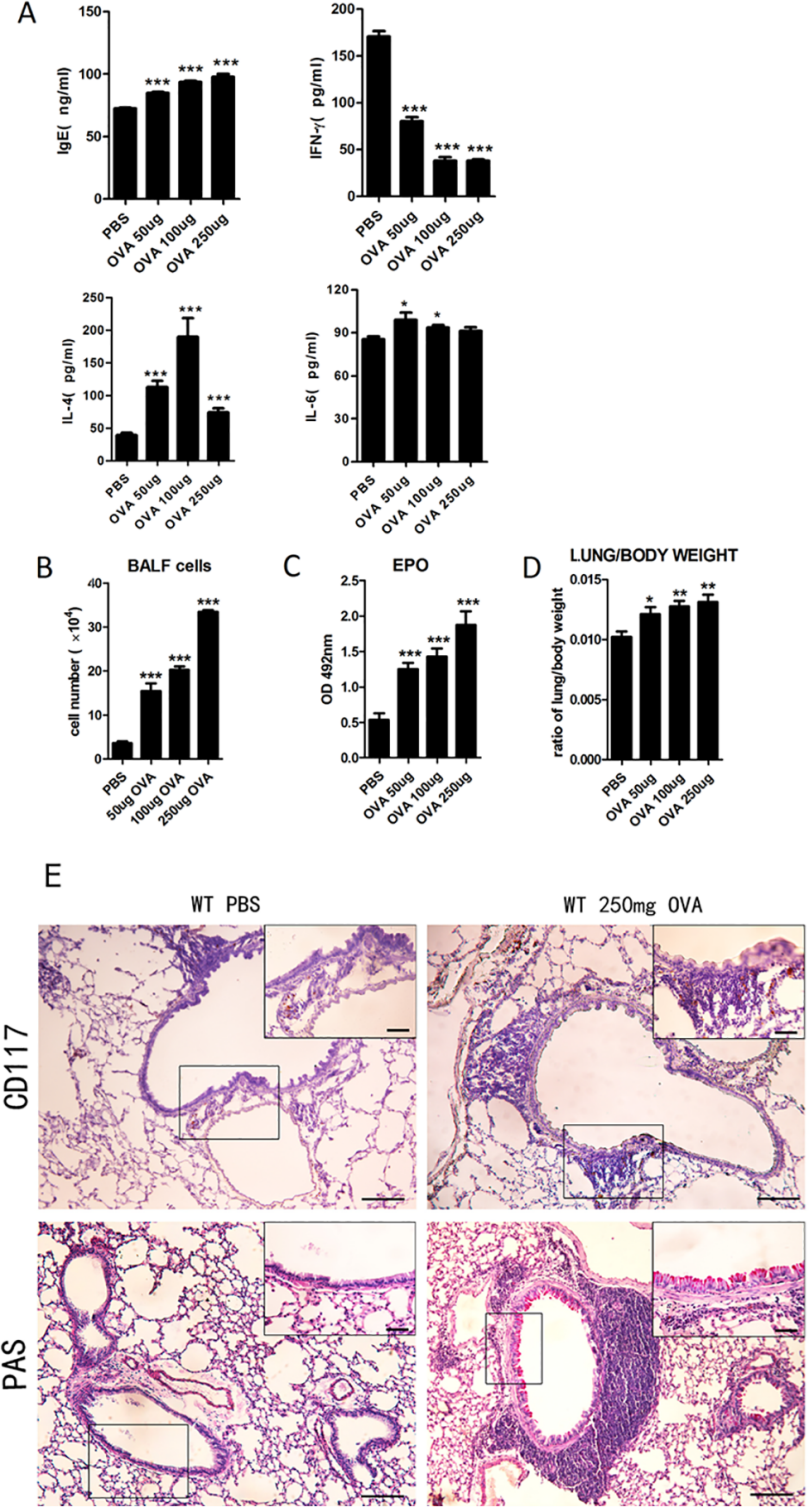
Detection of airway inflammation and morphological alterations following treatments with different concentrations of OVA in OVA-induced asthma mouse model. (A) ELISA analyses of the expression levels of IgE in the serum and IL-4, IL-6, and IFN-γ in the BALF following treatments with different concentrations of OVA in OVA-induced asthmatic mice. (B**)** The number of cells was quantified using a blood cell counting plate in the BALF for the different concentrations of OVA in OVA-induced asthma mice. (C**)** Eosinophil peroxidase activity was measured in the lung tissues of the asthmatic mice. (D) Lung edema was determined according to the lung/body weight data in OVA-induced asthma mice. (E) The staining of tissue for the detection of mast cells with anti-CD117 antibody in the lung tissues of the mice challenged with OVA. H&E staining of lung sections revealed the thickness of the alveolar walls, and PAS staining showed goblet cell hyperplasia along with mucus production in the lung tissues of the OVA-induced asthmatic mice. Lung sections were examined at the original magnification of 10× and at a higher magnification of 40×, as shown in the black square in the upper right corner of each picture. Scale bars: 100 μm in the panel. The data were normalized to the PBS sample data and are presented as the mean ± SEM (n = 6 animals for each genotype, as indicated by asterisks (* *P* < 0.05, ** *P* < 0.01, and *** *P* < 0.001).

To study inflammation in OVA-induced allergic asthma mice, the expression levels of IL-4, IL-6, IFN-γ in the BALF and IgE in the serum were determined by ELISA [18–22]. We found that there were increases in the levels of IgE, IL-4, and IL-6 in an OVA concentration-dependent manner, while the level of IFN-γ, which is a Th1-related cytokine, was decreased (Fig 1A). To investigate inflammatory cell invasion, we counted the number of BALF cells. The total number of infiltrating cells in the BALF was increased under the OVA challenge (Fig 1B). At the same time, eosinophil invasion, which is characteristic of allergic asthma, was determined according to eosinophil peroxidase (EPO) activity. The results showed that there was a marked increase in eosinophil activity, even at the low concentration of OVA (Fig 1C). To explore changes in the lung airway wall in OVA-induced allergic asthma mice, the lungs were weighed, and the morphological characteristics were observed. The results showed that the lungs of the OVA-induced mice increased in weight (Fig 1D). On the other hand, an increase in CD117-positive mast cells was detected in the investigation of mast cell invasion in the lung tissues, which is anther characteristic of asthma. Immunohistochemical analysis of the slices showed that the amount of CD117-positive mast cells in the mouse lungs was much higher following induction with OVA (Fig 1E). The number of PAS-stained cells in the bronchus of the lung was also increased sharply, and airway epithelial extensions along with mucus production were evident under a light microscope (Fig 1E). According to our results, 250 μg ovalbumin, which was the suitable concentration for the sensitized treatment in the allergic asthma mouse model, was used for the *Gpr97*-deficient asthma mouse model.

In order to detect the function of Gpr97 on immune cells involved in asthma, the expressions of Gpr97 on mRNA level were analyzed in lung tissues, mast cells and eosinophils using real-time PCR (S1 Table). After sensitization and challenge of OVA on mast cells, the expression of Gpr97 was increased almost twice as that of control (S1A Fig). Furthermore, its expressions in lung tissues were also higher in OVA-induced asthmatic mice than in control group (S1B Fig). The mRNA expression of Gpr97 was reduced in eosinophil with OVA challenge (S1C Fig). Then we would analyze the influence of Gpr97 on cytokines releasing and immune cells invasions in OVA-induced asthma in mice.

### *Gpr97* deficiency has no effect on antibody response or cytokine levels

**Fig 2:**
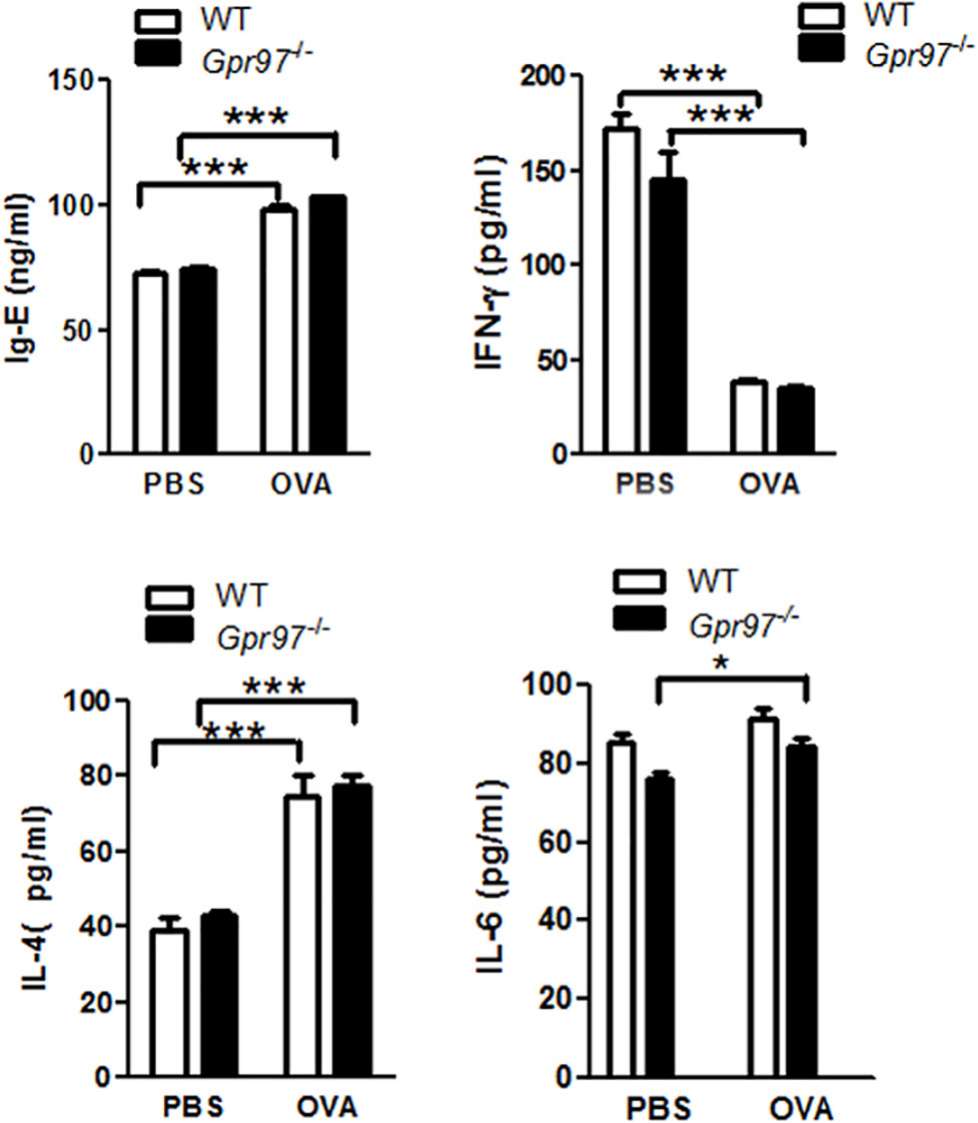
Comparisons of serum IgE and cytokine levels in BALF between WT and *Gpr97* ^*-/-*^ OVA-induced asthmatic mice. The levels of the cytokines IL-4, IL-6 and IFN-γ in the BALF and of IgE in the serum were measured after OVA induction by ELISA. The *t*-test was used to analyze significant differences of IL-4 and IFN-γ between WT and *Gpr97* ^***-/-***^ group. The altering of IL-6 was analyzed through an ANOVA+Tukey test. The data were normalized to the PBS sample data and are presented as the mean ± SEM (n = 6 animals for each genotype, as indicated by asterisks (* *P* < 0.05 and *** *P* < 0.001).

Airway inflammation and airway remodeling were best achieved by sensitization with 250 μg OVA. Hence, an asthma model was constructed in *Gpr97* ^*-/-*^ mice under the same conditions to determine whether Gpr97 affects allergic airway inflammation. According to our results, OVA challenge was also able to increase the secretion of IgE into the serum in the *Gpr97*-deficient mice, but no significant difference was observed in the WT mice. Then, we investigated whether Gpr97 has functions in the regulation of the inflammatory response and the differentiation of Th1/Th2 cells in OVA-induced allergic asthma mice by assessing serum IgE secretion and the expression of the Th1-related cytokine IFN-γ and the Th2-related cytokines IL-4 and IL-6 in the BALF. The results showed that there were increases in the expression levels of the Th2-related cytokines IL-4 and IL-6 and a decrease in the expression of the Th1-related cytokine IFN-γ in the OVA-challenged *Gpr97* ^*-/-*^ mice compared with the saline-treated deficient mice. However, no obvious differences in the levels of cytokines were found between the WT and *Gpr97* ^*-/-*^ mice following treatment with the same OVA concentration (Fig 2).

### No alteration in recruitment of inflammatory cells occurs in *Gpr97* ^*-/-*^ asthmatic mice

**Fig 3:**
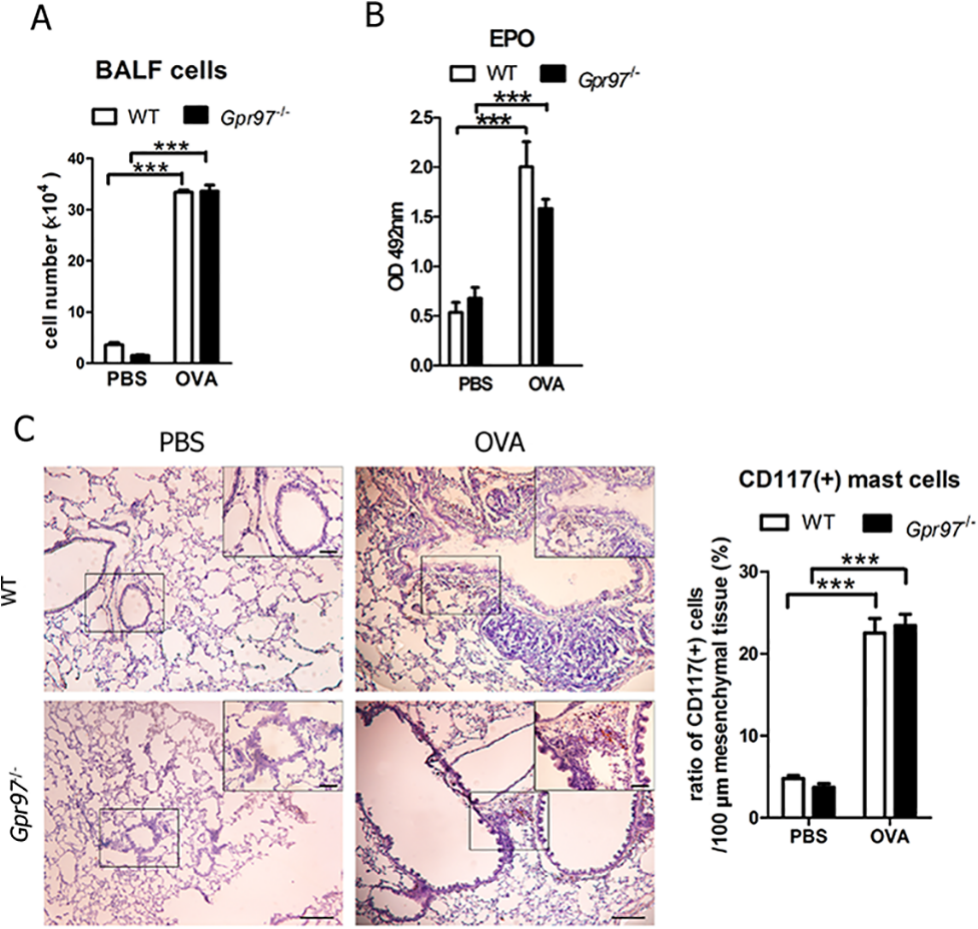
Inflammatory cell recruitment in lung tissues of WT and *Gpr97* ^*-/-*^ OVA-induced asthmatic mice. (A) The numbers of cells in the BALF were quantified for the WT and *Gpr97* ^***-/-***^ asthmatic mice following OVA induction using a blood cell counting plate. (B) Eosinophil peroxidase activities of eosinophils in the lung tissues were measured in OVA-induced asthma mice. (C) The number of CD117-positive mast cells that invaded the lung tissues of the WT and *Gpr97* ^***-/-***^ asthmatic mice were examined by light microscopy and counted with Image-Pro Plus 6.0 software. The lung sections were observed at 10× magnification and at a higher magnification of 40×, as shown in the black square in the upper right corner of each picture. Scale bars: 100 μm in the panel. The data were normalized to the PBS sample data and are presented as the mean ± SEM (n = 6 animals for each genotype, as indicated by asterisks (*** *P* < 0.001).

We next investigated whether Gpr97 has a role in recruiting inflammatory cells in the process of allergic asthma. As shown in Fig 3A and 3C, cells infiltrated into the lung parenchyma, and the total number of cells in the BALF were increased significantly in both the OVA-challenged WT and *Gpr97* ^*-/-*^ mice compared with the PBS-treated groups. However, there was no difference in BALF cell number between the OVA-induced WT and *Gpr97* ^*-/-*^ mice. Eosinophil invasion was observed in the WT mice after OVA challenge in our former experiment by the measurement of eosinophil peroxidase activity and number of Siglec-F^+^/CCR3^+^ eosinophil in BALF (Siglec-F, an early eosinophil development surface marker. CCR3, an eosinophil surface marker [23]). The same phenomenon was also detected in the *Gpr97*-deficient mice treated with OVA (Fig 3B, S2 Fig). Furthermore, immunohistochemistry revealed that CD117-positive mast cell invasion occurred in the lung tissues of the OVA-induced *Gpr97* ^*-/-*^ mice but that no invasion occurred in the control group of deficient mice (Fig 3C). The number of CD117-positive cells was exceedingly higher in both the OVA-challenged WT and *Gpr97* ^*-/-*^ mice than in the control mice. Although the eosinophils and mast cells were both increased in all OVA-treated mice, there were still no striking differences between the WT and *Gpr97* ^*-/-*^ mice. In brief, these results indicated that *Gpr97* deficiency did not affect inflammatory cell invasion during OVA-induced airway inflammation in our mouse models.

### Gpr97 does not affect airway conformation or mucus production in OVA-induced mice

**Fig 4:**
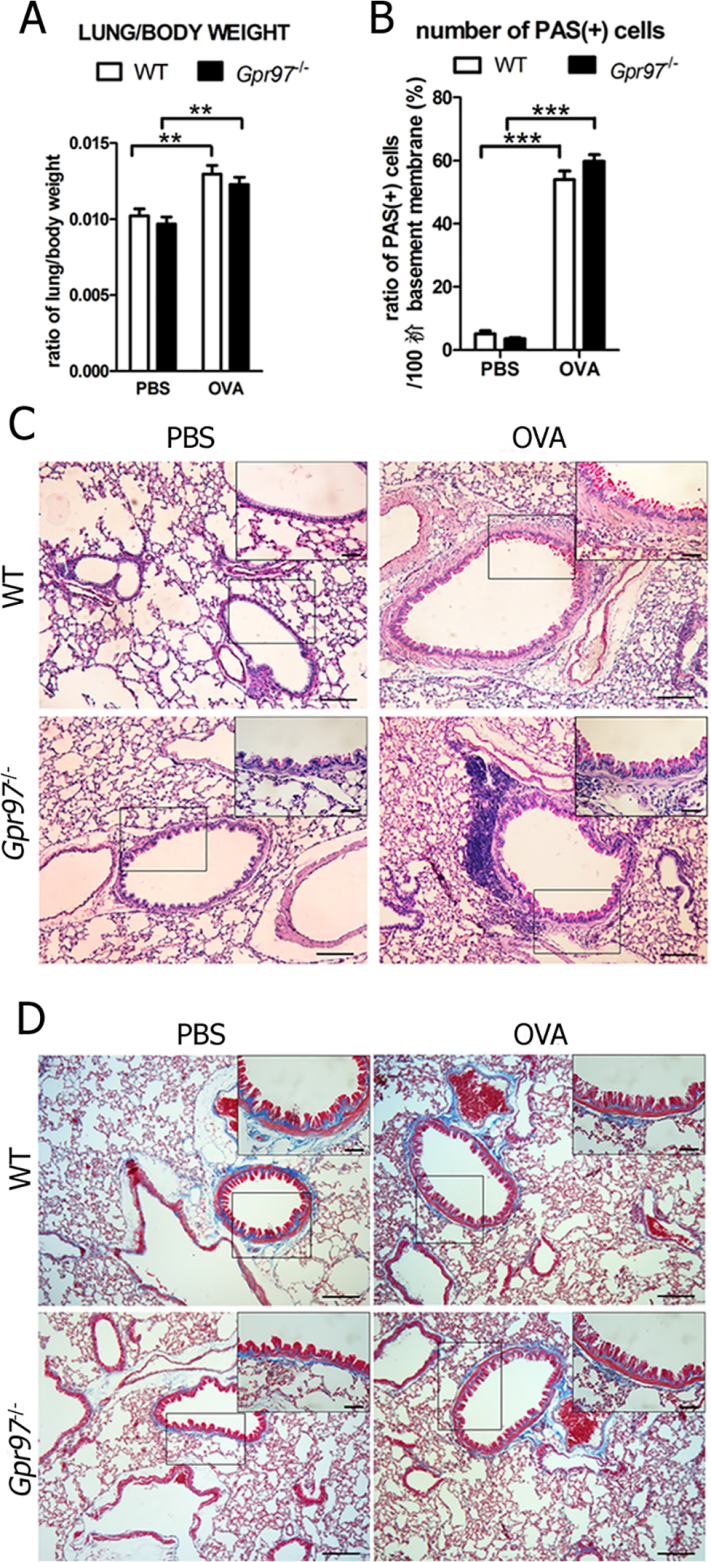
Airway remodeling of lung tissues in OVA-induced WT and *Gpr97* ^*-/-*^ asthmatic mice. (A) Lung edema following OVA challenge was determined according to the lung/body weight ratio. (B) Hyperplasia of the goblet cells along with mucus production were measured by PAS staining and examined by light microscopy at 10× magnification. The black square in the upper right corner of each picture shows the positively stained area at a higher magnification of 40×. Scale bars: 100 μm in the panel. (C) The ratios of PAS-positive goblet cells were counted using Image-Pro Plus 6.0 software. The data were normalized to the PBS sample data and are presented as the mean ± SEM (n = 6 animals for each genotype, as indicated by asterisks (** *P* < 0.01, and *** *P* < 0.001). (D) Masson’s trichrome staining was used to determinate the subepithelial fibrosis in lung tissues in OVA and saline challenge mice. The black square in the upper right corner of each picture shows the positively stained area at a higher magnification of 40×. In the pictures, cytoplasm is stained in red and collagen in blue.

Next, we evaluated the effects of Gpr97 on airway structural conformation and mucus hypersecretion in lung tissue, which are two additional characteristics of OVA-induced allergic asthma [24]. Lungs were weighed to analyze airway edema. The lung/body weight ratios were slightly increased in the WT and *Gpr97*-deficient mice following the OVA challenge compared to the saline challenge. However, no alterations in edema occurred in the OVA-induced *Gpr97* ^*-/-*^ mice (Fig 4A). In the lung tissues of the mice with allergic asthma, hyperplasia of the goblet cells, which are mostly mucus-producing cells, was observed in our previous experiment. At same time, a similar hyperplasia of the goblet cells was also observed under a light microscope in the OVA-treated *Gpr97*-deficient mice using the PAS special staining method (Fig 4B). The number of PAS-positive cells was determined by Soft Image-Pro Plus (Version 6.0), and the results showed a greater increase in goblet cells in the airway epithelial layer of the OVA-challenged mice regardless of whether they were *Gpr97*-deficient (Fig 4C). There was still no difference in goblet cell hyperplasia or mucus hypersecretion between the WT and *Gpr97* ^*-/-*^ allergic asthma mice. Then the subepithelial fibrosis in lung tissues were determinate using Masson’s trichrome staining. Although the deposition of extracellular matrix was increased with OVA challenge, but no obvious difference was found in *Gpr97*-deficient asthmatic mice (Fig 4D).

## Discussion

Allergen-induced airway inflammation is mainly characterized by an immune response driven by common allergens, such as mast cells, eosinophils, Th2 cells and B lymphocytes [22,25,26]. Among all of the types of allergens, including pollen, fungi, house dust mites and OVA, OVA is one of the most commonly used inducers for establishing animal models of allergic asthma [27]. In this study, OVA was used as an allergen to construct an airway inflammation mouse model. According to our results, the dysfunction of the airway and inflammation in the lung tissues were the most severe following sensitization with 250 μg OVA, which resulted in increases in the IgE antibody level, the release of Th2-related inflammatory factors, the invasion of lymphocytes, including eosinophils and mast cells, into lung tissues, airway remodeling and mucus hypersecretion. Hence, WT and *Gpr97*-deficient mice were challenged by OVA to establish allergic asthma mouse models under this condition.

In our study, we assessed the function of Gpr97 in OVA-induced asthma using *Gpr97*-deficient mice. Human GPR97 belongs to a family of adhesion GPCRs that have been confirmed to play critical roles in the central nervous system, the immune system and tumor formation [9,28]. *In vitro* experiments have shown that GPR97 can be activated first by a small-molecule compound, namely beclomethasone dipropionate (BDP), and that it then binds to the Go protein to activate downstream intracellular messengers [28]. BDP is commonly used in the clinical therapy of asthma to aid in the reduction of inflammation, although its molecular mechanism is still unclear [29]. Human GPR97 expressed in immune cells and lymphatic endothelial cells contributes to cytokine release and cell migration [10,11,28]. Thus, we inferred that it may play a regulatory role in the inflammatory response of asthma. In our mouse model of allergic asthma, the effect of Gpr97 on alterations in inflammatory cytokine levels was examined. We found that the deficiency in *Gpr97* did not affect the altering obviously in Th2-related cytokines, including IL-4 and IL-6, and the significant decrease in the Th1-related cytokine IFN-γ, suggesting that Gpr97 is not essential for T cell proliferation and differentiation in asthma. With regard to immune cell invasion into the lung tissues of the asthmatic mice, similar alterations were observed in the OVA-induced *Gpr97* ^*-/-*^ mice, such as a sharp increase in the total number of cells in the BALF.

In asthma, the major cell types that infiltrate the airway include eosinophils and mast cells, which are important sources of numerous cytokines that are released to augment the inflammation of lung tissue [30]. Because GPR97 was overexpressed in the mast cells, eosinophils, neutrophils and other immune cells, alterations in the levels of eosinophils and mast cells, which are two of the important types of immune cells, were examined further in our asthma mouse model. Although a sharp increase in the mRNA expression of Gpr97 in mast cells and lung tissue were found after OVA stimulation (S1 Fig), no obvious change occurred after *Gpr97* knockout in asthmatic mice. Moreover, the expression of Gpr97 was reduced in eosinophil in OVA-induced asthmatic mouse, but *Gpr97* deficiency did not impair the infiltration of eosinophils into the bronchial epithelium according to our results. Eosinophils and mast cells have been confirmed to affect the proliferation and desquamation of epithelial cells and the formation of goblet cells; thus, they may influence the barrier function of epithelial cells [31,32]. The secretion of mucus by goblet cells leads to the exacerbation of the airway remodeling phenotype and irreversible or partially reversible airflow obstruction [22,24,33]. PAS staining showed that the number of goblet cells in the airway epithelium increased markedly following OVA treatment. These features of airway remodeling were also found in the *Gpr97* ^*-/-*^ allergen-induced asthmatic mice to a similar extent. Thus, Gpr97 may not be required in the regulation of airway caliber or mucus production in OVA-induced asthma.

In asthma, the serum IgE level is increased following allergen stimulation, and it then binds to the high-affinity receptor FcεRI, which is mainly expressed in mast cells, eosinophils and basophils [1]. This activity triggers the activities of mast cells, including the release of some cytokines, the regulation of inflammation and airway remodeling. Moreover, several studies have suggested that some cytokines produced by mast cells, including IL-4 and IL-6, can also stimulate the proliferation and differentiation of activated B cells [34,35]. These cytokines enhance IgE production in B cells and cause allergic asthma deterioration. We found that Gpr97 was not involved in the increase in IgE in the sera of the mice. The levels of IL-4 and IL-6 and mast cell invasion were not altered in the *Gpr97* ^*-/-*^ asthmatic mice, indicating that Gpr97 might not take part in mast cell activation following IgE stimulation or cytokine production in the process of asthma induction.

Gpr97 appears to be highly expressed in immune cells and may be involved in inflammatory responses, including allergic airway inflammation. The deficiency of *Gpr97* did not alter inflammation in our OVA-induced asthma mouse model. All of the findings of our study, including the lack of significant alterations in the release of cytokines and IgE, immune cell invasion and airway remodeling in the lung tissues of the *Gpr97*-deficient mice, indicate that Gpr97 might not play an important role in the inflammatory response in allergic asthma. However, it is essential for the regulation of the development of B cells, which produce IgE during the asthma process [12]. Hence, other immune cells activated during the initial stages of asthma, such as B cells, dendritic cells or macrophages, may have prominent roles following *Gpr97* deficiency in the asthma mouse model. Other chronic asthma models should also be assessed to clarify the function of Gpr97 in the allergic inflammatory response. Nevertheless, further investigations need to address the contribution of Gpr97 in airway inflammation in the lung. Moreover, other members of adhesion GPCRs, such as Gpr56 and Gpr114, might be involved in the development of OVA-induced asthma according to our results. Although no obvious altering of mRNA levels of Gpr114 and Gpr56 were found in lung tissues, they were both reduced in mast cells and eosinophils after OVA challenge in mice (S1 Fig). So in adhesion GPCR family, Gpr97 showed most influenced with OVA challenge and the other member was Gpr114. Gpr114 might also be required in OVA-induced asthma development and its function in which should be further investigated.

## Supporting Information

S1 Fig The mRNA expression levels of Gpr97, Gpr56 and Gpr114 with OVA challenge on cell and lung tissue of mice. (A) The determination of mRNA expression of Gpr97 on OVA-induced mast cell. Before OVA challenge, mast cells were activated with total serum-IgE from OVA-induced mice overnight. (B) The mRNA levels of Gpr97, Gpr114 and Gpr56 in lung tissues of OVA-induced asthmatic mice. (C) CD117 positive mast cells were selected from BALF using flow cytometry from WT mice after saline or OVA challenge. Expressions of Gpr114 and Gpr56 were detected in mRNA levels using Real-time PCR. (D) The CCR3/Siglec-F positive eosinophil were purified from BALF of saline or OVA-induced mice using flow cytometry method separately. mRNA expression levels of the genes encoding Gpr97, Gpr114 and Gpr56 were detected using Real-time PCR. Data shown as mean ± SEM (n = 6, * *P* < 0.05, ** *P* < 0.01, and *** *P* < 0.001).
(TIF)

S2 Fig The number of eosinophil infiltration in BALF with OVA challenge in mice. (A) The ratio of Siglec-F^+^/CCR3^+^ eosinophil in BALF using flow cytometry. Antibodies of Siglec-F and CCR3 were used to confirmed eosinophil in BALF with saline or OVA challenge in mice. (B) The total number of Siglec-F^+^/ CCR3^+^ eosinophil infiltration in BALF was counted according to the ratio of eosinophil in BALF and the number of total cells in BALF. Data shown as mean ± SEM (** *P* < 0.01).
(TIF)

S1 Table The sequences of the primers for Real-time PCR. (DOCX)
