# Supplementary figures and images for: Gpr97 Is Dispensable for Inflammation in OVA-Induced Asthmatic Mice
Source: PLoS One. 2015 Jul 1;10(7):e0131461. doi: 10.1371/journal.pone.0131461 (PMC4489018; doi:10.1371/journal.pone.0131461)

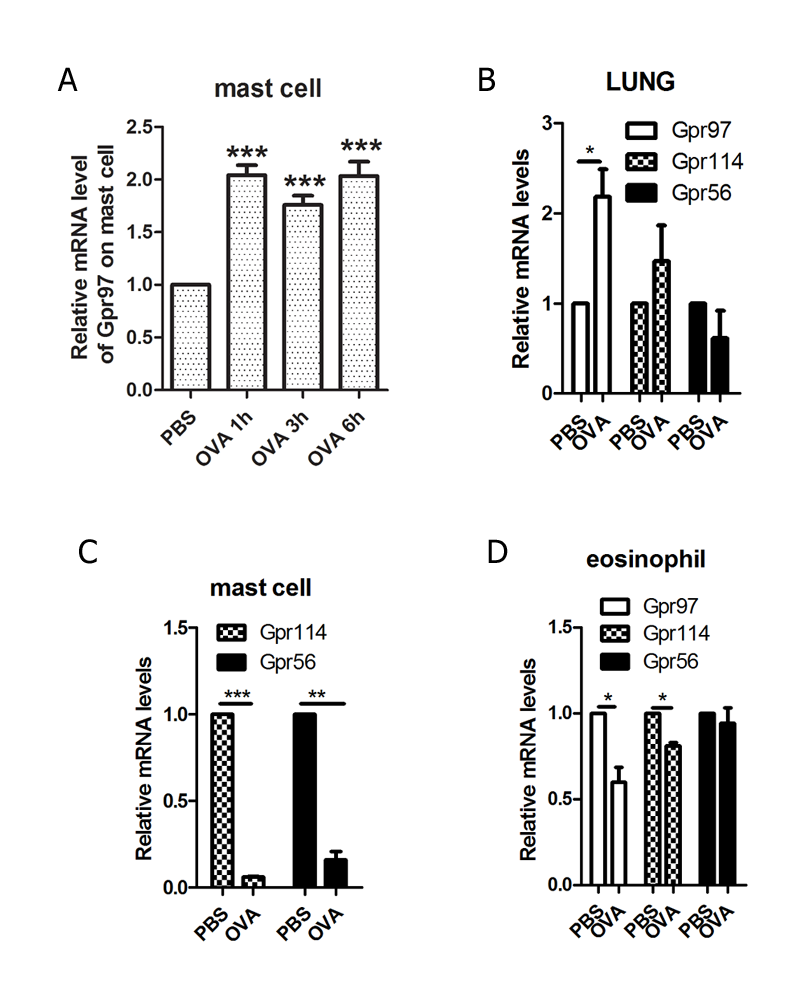

Supplement: S1 Fig — (A) The determination of mRNA expression of Gpr97 on OVA-induced mast cell. Before OVA challenge, mast cells were activated with total serum-IgE from OVA-induced mice overnight. (B) The mRNA levels of Gpr97, Gpr114 and Gpr56 in lung tissues of OVA-induced asthmatic mice. (C) CD117 positive mast cells were selected from BALF using flow cytometry from WT mice after saline or OVA challenge. Expressions of Gpr114 and Gpr56 were detected in mRNA levels using Real-time PCR. (D) The CCR3/Siglec-F positive eosinophil were purified from BALF of saline or OVA-induced mice using flow cytometry method separately. mRNA expression levels of the genes encoding Gpr97, Gpr114 and Gpr56 were detected using Real-time PCR. Data shown as mean ± SEM (n = 6, * P < 0.05, ** P < 0.01, and *** P < 0.001). (TIF) [file pone.0131461.s001.tif]

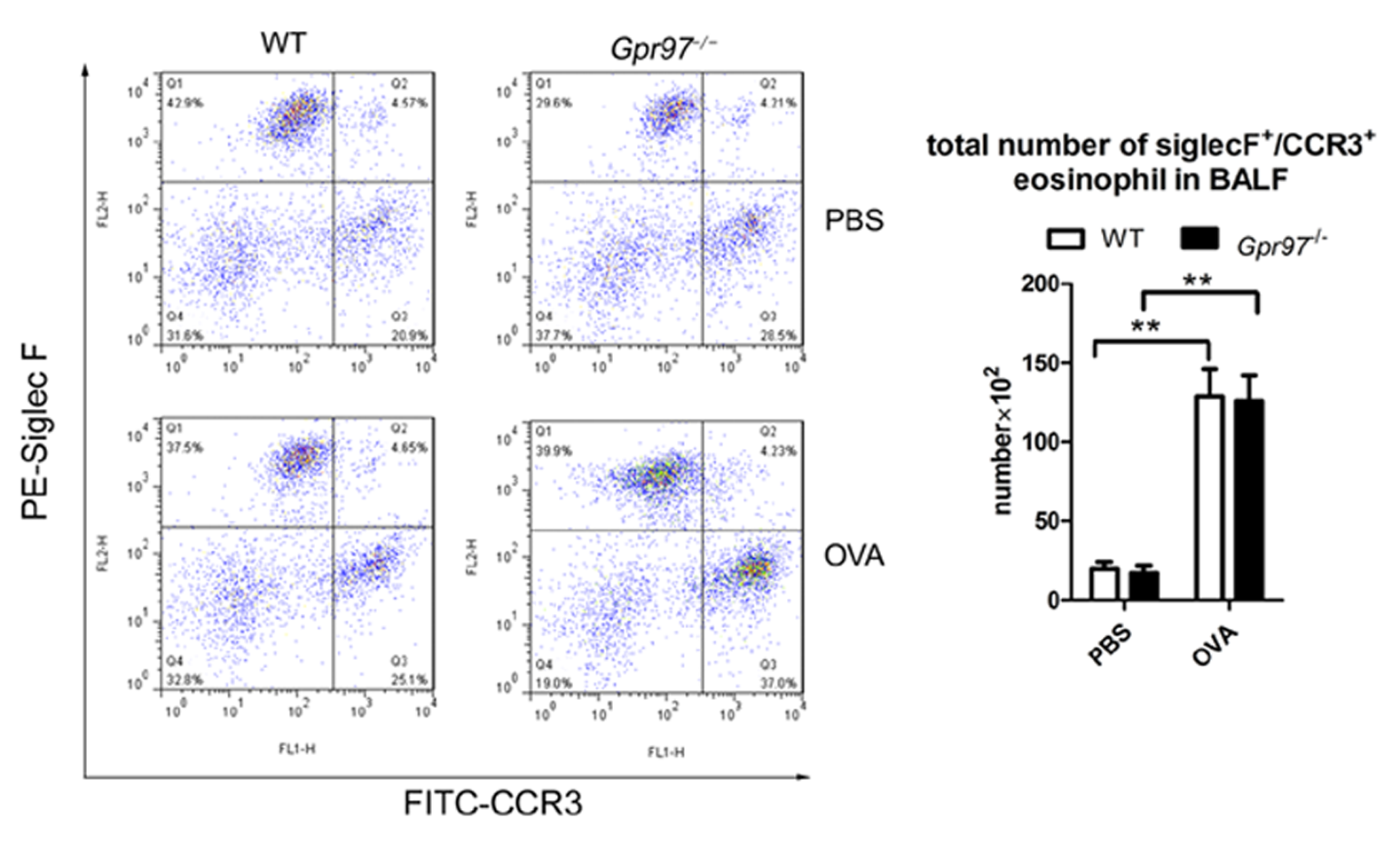

Supplement: S2 Fig — (A) The ratio of Siglec-F+/CCR3+ eosinophil in BALF using flow cytometry. Antibodies of Siglec-F and CCR3 were used to confirmed eosinophil in BALF with saline or OVA challenge in mice. (B) The total number of Siglec-F+/ CCR3+ eosinophil infiltration in BALF was counted according to the ratio of eosinophil in BALF and the number of total cells in BALF. Data shown as mean ± SEM (** P < 0.01). (TIF) [file pone.0131461.s002.tif]
