# Supplementary material for: Gpr97 Is Dispensable for Inflammation in OVA-Induced Asthmatic Mice
Source: PLoS One. 2015 Jul 1;10(7):e0131461. doi: 10.1371/journal.pone.0131461 (PMC4489018; doi:10.1371/journal.pone.0131461)
Supplement: S1 Table — (DOCX) [file pone.0131461.s003.docx]

| Gene name | Primers (5’- 3’) |
| --- | --- |
| *Gpr97* | forward: caccttcgacttgaatgacactgctc |
|  | reverse: tgctgatgttctggatcaatgcctt |
| *Gpr114* | forward: CTTTCAGGATGACCGGAACTC |
|  | reverse: CAGACTCCGATTATGCCAGAAG |
| *Gpr56* | forward: CTGCGGCAGATGGTCTACTTC |
|  | reverse: ATAGTGGAGGGTGCTCTGTTG |
| *β-actin* | forward: GTACGCCAACACAGTGCTG |
|  | reverse: CGTCATACTCCTGCTTGCTG |

**S3 Table. The sequences of the primers for Real-time PCR.**

These primers were used for detecting the mRNA levels of some adhesion GPCRs by Real-time PCR in S2 figure.
